# Fiber-Optimized Restoration: A Novel Approach to Strengthening Endodontic Posts With Dentapreg®

**DOI:** 10.7759/cureus.79806

**Published:** 2025-02-28

**Authors:** Sonali Bansal, Gaurav Patri, Neelanjana Majee, Ishika Chatterjee, Geonath Immanuel

**Affiliations:** 1 Conservative Dentistry and Endodontics, Kalinga Institute of Dental Sciences, Kalinga Institute of Industrial Technology (KIIT) Deemed to be University, Bhubaneswar, IND

**Keywords:** apexification, dentapreg ufm, endodontic treatment, fiber-reinforced composite, open apices

## Abstract

This case report discusses the management of a 42-year-old female patient with mature permanent teeth exhibiting open apices and significant structural damage due to trauma. Following incomplete endodontic treatment, teeth 21 and 22 presented with Ellis Class III fractures and open apices, while tooth 11 had a closed apex with an ill-defined radiolucency. Treatment involved apexification using Biodentine (Septodont, Saint-Maur-des-Fossés, France), laser-assisted disinfection, and reinforcement with fiber-reinforced composites (Dentapreg® Ultra Fine Mesh (UFM), Advanced Dental Material, Brno, Czech Republic). Post-endodontic restoration included porcelain-fused-to-metal crowns. The integration of modern materials and techniques, such as laser disinfection and fiber-reinforced posts, resulted in successful structural reinforcement and apical closure, highlighting the effectiveness of contemporary endodontic approaches in treating complex cases.

## Introduction

Open apex in mature permanent teeth caused by trauma, excessive orthodontic forces, chronic inflammation, or sometimes idiopathic factors presents significant challenges in endodontics, complicating standard treatments and demanding innovative approaches for successful outcomes. Causes of open apex include trauma that disrupts root development, leading to an incomplete apex, and pulpal necrosis due to untreated dental caries or repeated procedures [1]. Congenital anomalies like dentin dysplasia, odontodysplasia, and systemic conditions such as hypophosphatasia can also interfere with normal root formation, resulting in an open apex [2].

Treatment for non-vital teeth with open apices focuses on disinfecting the root canal and promoting apical closure. Apexification, a traditional method, induces a calcific barrier at the apex. It starts with thorough root canal sterilization to prevent reinfection and create a favorable environment for hard tissue formation. Calcium hydroxide has long been recognized for its antibacterial properties and its capacity to promote hard tissue formation [3]. However, newer materials like mineral trioxide aggregate (MTA) and Biodentine (Septodont, Saint-Maur-des-Fossés, France) have shown superior outcomes, offering better seal and more durable apexification. Recent advancements include bioactive glass and other bioceramic materials that enhance regenerative potential [2].

Regenerative endodontic procedures (REPs) are emerging as a promising alternative for managing vital immature permanent teeth with open apices [4]. REPs aim to restore dental pulp vitality and promote natural root development using tissue engineering principles, including stem cells, growth factors, and scaffolds. This approach represents a significant shift from traditional techniques, offering potential for the true biological healing and regeneration of dental pulp and root structures [3].

Lasers have revolutionized root canal sterilization, offering enhanced disinfection. Lasers such as the erbium-doped yttrium aluminum garnet (Er:YAG) and diode lasers can penetrate deep into the dentinal tubules, reaching depths of approximately 1 mm. The thermal effect generated by lasers aids in disinfection by denaturing bacterial proteins and disrupting cell walls. Additionally, lasers can activate photosensitizers in photoactivated disinfection (PAD), producing reactive oxygen species lethal to microorganisms, making lasers highly effective in improving clinical outcomes [5,6].

The enduring success of the endodontically treated teeth relies significantly on the importance of post-endodontic restoration. Without adequate restoration, teeth are at higher risk of failure and extraction. Timely and appropriate restoration, often involving root canal therapy, is essential for durability [7]. There are a large variety of materials and techniques to restore structurally compromised endodontically treated teeth. Historically, post and core restorations have been employed in endodontically treated teeth to restore structural integrity and function. The main purpose of the post and core is to restore the lost coronal tooth structure, ensuring sufficient retention and resistance for the final crown, which will ultimately restore the tooth's function and aesthetics. A post and core restoration is recommended for teeth with significant crown loss, a high risk of cervical fracture, loss of two proximal surfaces, or insufficient retention due to a shortened structure, provided the periodontal and periapical conditions are favorable. Fibers associated with composites can potentially counteract the adverse effects of resins' polymerization shrinkage and the consequent stress transferred to the composites and the remaining dental hard tissues. Simultaneously, they can promote improvement in the physical properties of composite restorations. Fiber-reinforced composite (FRC) technology, particularly Dentapreg® Ultra Fine Mesh (UFM) (Advanced Dental Material, Brno, Czech Republic), plays a vital role in reinforcing mutilated teeth, offering enhanced strength and flexibility. FRC significantly increases the fracture resistance of endodontically treated teeth, providing a conservative alternative to traditional crowns [8]. In the case of ultra-fine membrane (Dentapreg® UFM), the threads of fibers are loosely woven to provide flexibility and stretchability when adapted to a complex surface. The strips with unidirectionally oriented fibers (Dentapreg® Splinting Fiber-Unidirectional Unit (SFU) and Posterior Fiber-Reinforced Unit (PFU)) allow for dimensional change of their cross-section due to the limited flow of the impregnating resin matrix and corresponding movement of the individual fibers.

This case report introduces a novel technique of using Dentapreg® fibers to wrap around the fiber post and extend coronally to reinforce both the post and the core while also improving post adaptation to the aberrant canal wall with minimal canal preparation.

## Case presentation

**Figure 1:**
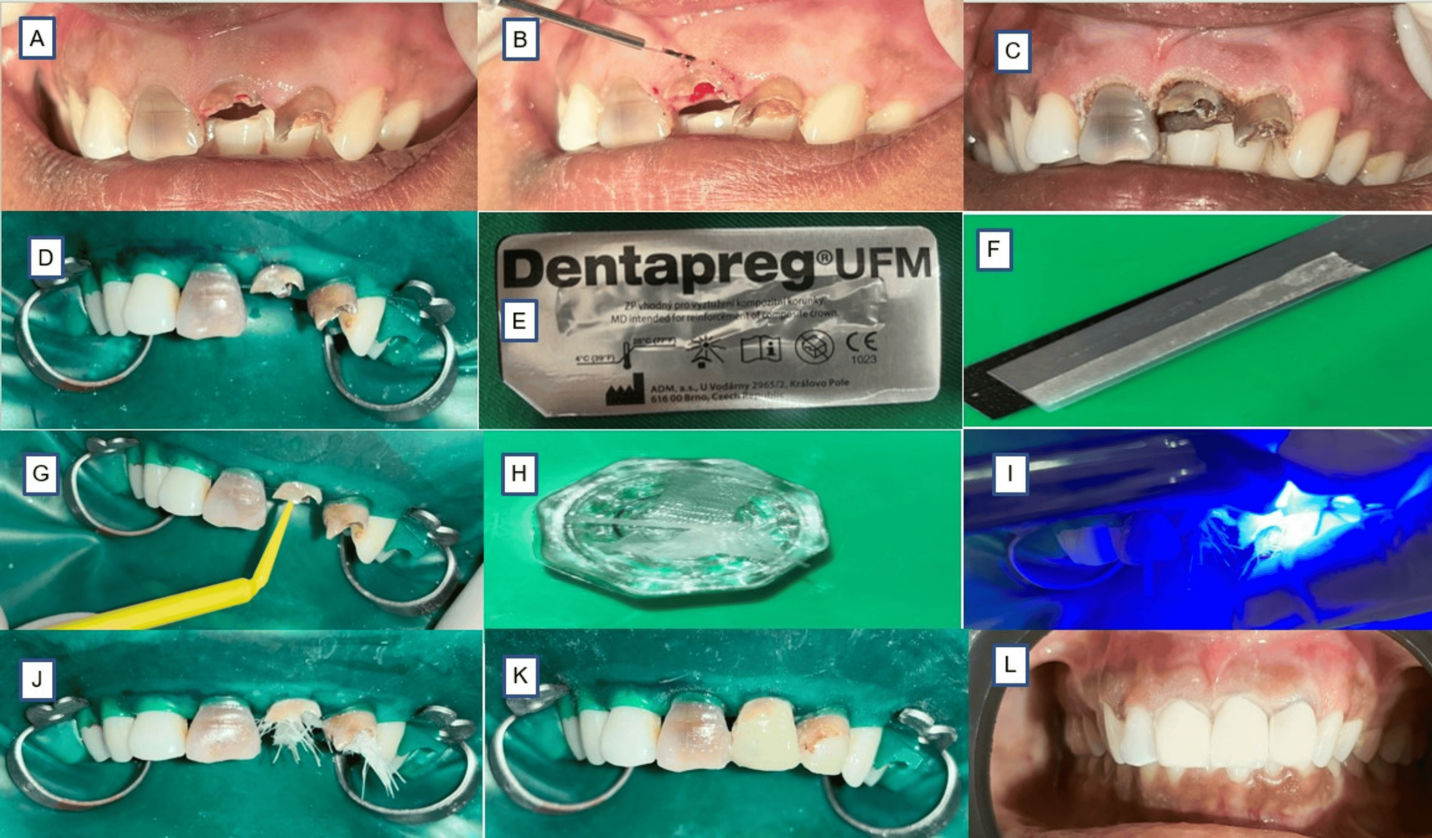
Clinical presentation (A) Pre-op. (B) Laser-assisted gingivectomy. (C) Immediate post-op following gingivectomy. (D) Pre-treatment isolation. (E) Dentapreg® UFM fibers. (F) 2 mm×10 mm Dentapreg® UFM fiber sheet. (G) Single bond adhesive (3M/ESPE) applied to all surfaces. (H) Dentapreg® UFM fibers wrapped the fiber post. (I) Dentapreg® fiber-wrapped post placed inside the canal using dual cure resin cement and cured. (J) Fibers left extended beyond the canal. (K) Core build-up. (L) PFM crown cementation after one month PFM: porcelain-fused-to-metal

**Figure 2:**
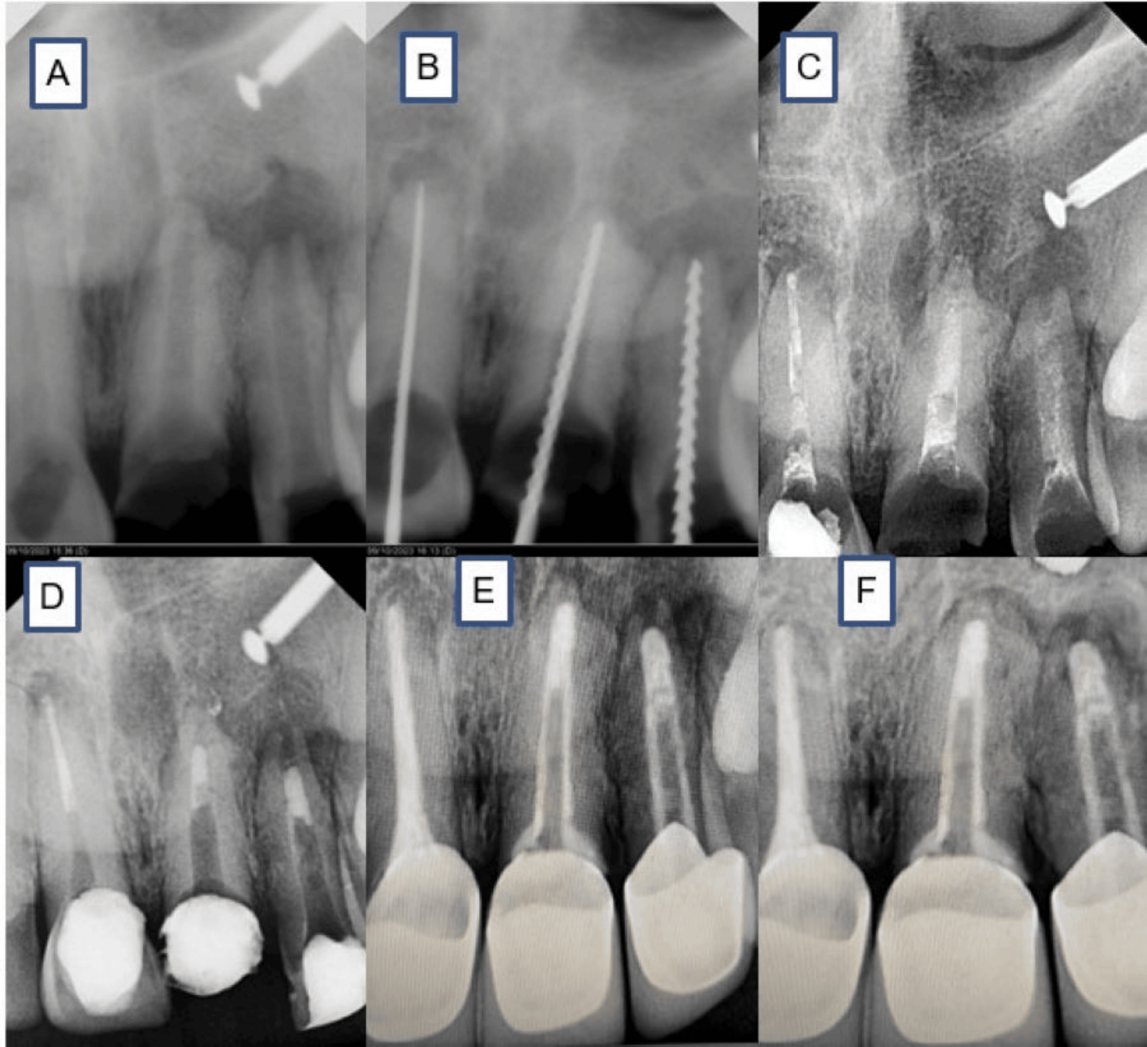
Radiographic representation (A) Pre-op. (B) Working length determination. (C) Ca(OH)2 dressing placed. (D) A-PRF+Biodentin placed. (E) One-month post-op. (F) Nine months follow-up Ca(OH)2: calcium hydroxide; A-PRF: advanced platelet-rich fibrin

A 42-year-old woman visited the Department of Conservative Dentistry and Endodontics, reporting pain and swelling in the upper front tooth area as her primary concern. The patient had a history of facial trauma from a bike accident 19 years ago, after which she received incomplete endodontic treatment for teeth 21 and 22. During the clinical assessment, teeth 21 and 22 were diagnosed with Ellis Class III fractures, and tooth 11 was identified with an Ellis Class IV fracture, accompanied by discoloration (Figure 1A). Radiographs showed radiolucent indentations near teeth 21 and 22 with root tip shortening and open apices, while tooth 11 had a closed apex with an ill-defined radiolucent area. Given the minimal remaining tooth structure in teeth 21 and 22, the restorative prognosis was unfavorable (Figure 2A).

The patient was informed about and presented with multiple treatment alternatives. She opted against extraction or surgical procedures. The treatment plan focused on saving teeth 11, 21, and 22, including re-treatment of teeth 21 and 22 with apexification and reinforcement using FRC resin with fiber-reinforced posts. Tooth 11 would undergo similar reinforcement following endodontic therapy. The patient consented to the proposed treatment plan, understanding the associated risks.

Treatment

The treatment plan was structured into two stages: stage 1 focused on apexification, while stage 2 involved the fabrication of the restoration.

Stage 1: Apexification

Local anesthesia was administered, and teeth were isolated with a dental dam. With the aid of a dental operating microscope (M320 F12, Leica Microsystems, Wetzlar, Germany), a standard access cavity was created using an Endo Access bur (A0164, Dentsply, Yverdon-les-Bains, Switzerland) and a no. 330 carbide bur. The working lengths were established using K-files (Dentsply Maillefer, Ballaigues, Switzerland) in conjunction with an electronic apex locator (Coltene, Altstätten, Switzerland) and were further verified through radiographic imaging (Figure 2B). The canals were shaped using hand files in a crown-down technique up to an apical size of 80, with intermittent irrigation using a 3.5% sodium hypochlorite (NaOCl) solution. Disinfection was performed with E2-14 EZ endo tips (BIOLASE, Foothill Ranch, CA, USA) in a sterilized laser handpiece using the diode laser disinfection phase. The laser tip was moved in a circular motion along the canal walls at a rate of 1 mm per second, in order to achieve thorough disinfection.

Calcium hydroxide was placed using a Lentulo spiral coated with the paste, inserted into the canal with a pumping motion to effectively fill the entire length, ensuring the material reaches the apex while removing excess with a sterile paper point or cotton swab, and used as an inter-appointment medication for a duration of seven days (Figure 2C), and the access cavity was temporarily sealed using Teflon tape and Cavit (3M, St. Paul, MN, USA). Gingivectomy was performed to enhance the crown height of teeth 11, 21, and 22 (Figure 1B, 1C). Since the keratinized gingiva was of sufficient width and the pocket depth measured 3 mm, a gingivectomy of up to 2 mm was planned using a 940 nm diode laser (BIOLASE, Foothill Ranch, CA, USA), set to operate at 3.25 W in pulse mode (20 ms pulse duration, 20 ms pulse interval) with a 400 micron fiber in contact mode.

During the second appointment, the acute symptoms had resolved, and the temporary filling was carefully removed. The canals were flushed with NaOCl to eliminate any residual calcium hydroxide. The apex position was determined for the final placement of Biodentine, using a stainless steel endodontic plugger connected to an electronic apex locator, with the position confirmed through radiographic imaging. The root canals were dried with sterile paper points, and advanced platelet-rich fibrin (A-PRF) was prepared from 5 mL of the patient's blood to be used as an apical barrier.

Biodentine was introduced into the canal using a curved needle and carefully compacted toward the apex with a root canal plugger under magnification from an endodontic microscope. To enhance adaptation, ultrasonic vibration was applied indirectly by placing an ultrasonic tip on the plugger, ensuring that 3 mm of the apical third of the root canal was filled with Biodentine (Figure 2D). In tooth 11, the apical 5 mm was obturated with gutta-percha. Due to the oval shape and flared anatomy of the canal, additional space was observed around the fiber post. To resolve this, an alternative approach was implemented: the fiber post was treated with a silane coupling agent for one minute using a disposable micro brush, followed by gentle air-drying for five seconds (Figure 1H). A 2-mm-wide and 10-mm-long segment of Dentapreg® UFM fiber was prepared (Figure 1E, 1F). A single-step adhesive system was applied to the fiber post, and the Dentapreg® UFM was wrapped around it before being light-cured for 10 seconds.

Stage 2: Restoration

The root canal walls and remaining dentin surfaces were etched with 37% phosphoric acid for 15 seconds, rinsed with water for 30 seconds, and air-dried gently. An adhesive system was applied, and the Dentapreg® UFM was coated with a single bond adhesive (3M/ESPE, St. Paul, MN, USA). A dual-cure resin cement (3M Relyx, St. Paul, MN, USA) was used to bond the fiber post wrapped in Dentapreg® UFM. The Dentapreg® was folded and inserted into the canal to maximize the reinforcement material while minimizing cement usage. It was extended 2 mm beyond the canal and light-cured for 20 seconds using a light-emitting diode (LED) curing unit (Figure 1G, 1I, 1J). Finally, the post was coated with a flowable composite (3M/ESPE), and the coronal restoration was completed using a packable resin composite (Figure 1K, 1E).

**Figure 3:**
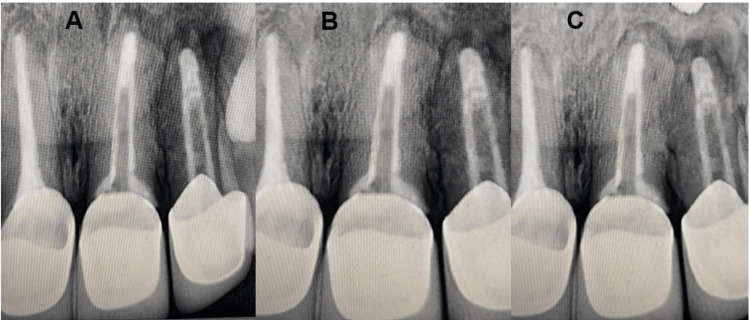
Radiographic follow-up (A) Three months follow-up. (B) Six months follow-up. (C) Nine months follow-up

Tooth preparation was done for a porcelain-fused-to-metal (PFM) crown, followed by the cementation of the PFM crown (Figure 1L). The patient was scheduled for follow-up evaluations at intervals of three, six, and nine months (Figure 3A, 3B, 3C).

This comprehensive approach, incorporating advanced materials and techniques, achieved a successful outcome in the management of mature permanent teeth with open apices and significant structural damage.

## Discussion

The presented case highlights the complexities and challenges associated with treating mature permanent teeth with open apices, especially when compounded by significant structural damage and a history of trauma. The successful management of such cases relies heavily on meticulous disinfection, proper material selection, and reinforcement of the remaining tooth structure. This case report illustrates the application of modern endodontic techniques and materials to address such challenges effectively.

Open apices in mature teeth are often the result of trauma or pulpal necrosis, leading to incomplete root development [9]. In the presented case, a history of facial trauma and incomplete endodontic treatment contributed to the compromised state of teeth 21 and 22, with an Ellis Class III fracture and wide open apices. The difficulty in treating such cases lies in achieving effective disinfection and promoting apical closure, which are crucial for long-term success [1].

Apexification aims to create an apical barrier in non-vital teeth with open apices. Traditionally, calcium hydroxide has been used due to its antimicrobial properties and ability to induce hard tissue formation [8]. However, recent advancements have introduced materials such as MTA and Biodentine, which offer superior outcomes. MTA and Biodentine facilitate the formation of a high-quality dentin bridge and provide a more reliable seal compared to calcium hydroxide [2]. Biodentine, in particular, has the advantage of faster setting time, which can be beneficial in clinical practice.

In this case, Biodentine with PRF was utilized to establish an apical barrier. Biodentine's ability to set quickly and form a durable barrier aligns with current best practices for apexification. This choice is supported by the literature, which highlights the superior performance of Biodentine in inducing apical closure and promoting healing [10,11]. A modern approach involves using PRF as an apical matrix membrane, leveraging its advantages over other materials. PRF is an autologous fibrin matrix enriched with platelets and leukocyte cytokines, which are progressively released over 7-11 days as the fibrin degrades. This gradual release supports rapid angiogenesis and efficient fibrin remodeling, aiding in optimal healing. PRF also acts as a mitogen, stimulating the proliferation of osteoblasts, gingival fibroblasts, and periodontal ligament cells. Additionally, it releases crucial growth factors such as platelet-derived growth factors (PDGF) and transforming growth factors (TGF), further promoting tissue regeneration. Most importantly, PRF is fully autologous, making it highly biocompatible compared to other materials used in apexification procedures, particularly in cases with wide-open apices [12].

The use of lasers, such as the Er and diode lasers, has enhanced root canal disinfection by penetrating deep into dentinal tubules and eradicating bacteria and biofilms more effectively than traditional irrigation techniques [13]. In this case, the diode laser was employed for disinfection, providing a significant advantage by enhancing the removal of bacterial contaminants and improving the overall cleanliness of the canal system. This approach aligns with current evidence suggesting that laser disinfection contributes to better clinical outcomes [14].

The use of FRCs has become increasingly common in the reinforcement of endodontically treated teeth. FRCs, such as those made from polyethylene or glass fibers, provide dentin-like properties and enhance the fracture resistance of weakened teeth [15]. The use of Dentapreg® UFM in this case for reinforcing teeth with extensive damage illustrates the benefits of modern materials in restoring structural integrity. FRCs not only improve biomechanical performance but also offer aesthetic advantages [16].

The restoration of endodontically treated teeth is critical for ensuring long-term success and functionality. The choice of post and core materials, along with the final coronal restoration, significantly impacts the durability of the treatment. In this case, the combination of fiber-reinforced posts and a PFM crown was employed to provide both structural support and aesthetic enhancement. Studies have demonstrated that adequate post-endodontic restoration reduces the risk of tooth failure and improves clinical outcomes [17].

## Conclusions

This case report highlights the successful application of modern endodontic techniques and materials in managing mature permanent teeth with open apices and significant structural damage. The use of Biodentine for apexification, laser disinfection, and FRCs for reinforcement exemplifies the integration of advanced approaches to achieve favorable outcomes. The comprehensive treatment plan not only addressed the immediate clinical challenges but also laid the foundation for long-term success and functionality.
